# Supplementary material for: Statistical Thermodynamics of Irreversible Aggregation: The Sol-Gel Transition
Source: Sci Rep. 2015 Mar 9;5:8855. doi: 10.1038/srep08855 (PMC4352918; doi:10.1038/srep08855)
Supplement: Supplementary Information — Derivations [file srep08855-s1.pdf]

Supplementary Information

Statistical Thermodynamics of Irreversible Aggregation and Gelation

Themis Matsoukas
Department of Chemical Engineering, Pennsylvania State University,
University Park, PA 16802

DERIVATIONS

A. On the mean kernel $\bar{k}_{\mathbf{n}}$ and the transition probability $P_{\mathbf{n}'\to\mathbf{n}}$

The mean kernel $\bar{k}_{\mathbf{n}}$ is the average over all pairs of distribution $\mathbf{n}$. For a distribution of the (M, N) ensemble, it is given by

$$\bar{k}_{\mathbf{n}} = \frac{1}{N(N-1)} \sum_{i=1}^{\infty} \sum_{j=1}^{\infty} n_i (n_j - \delta_{ij})\, k_{ij}. \tag{S1}$$

In this form the summation treats pairs as ordered such that all pairs off the diagonal ($i \neq j$) appear twice and those on the diagonal once. The summation can be re-indexed to treat pairs as unordered: cover the $i \times j$ domain up to the diagonal:

$$\bar{k}_{\mathbf{n}} = \frac{2}{N(N-1)} \sum_{i=1}^{\infty} \sum_{j=1}^{i} \frac{n_{i-j}(n_j - \delta_{i-j,j})}{1+\delta_{i-j,j}}\, k_{i-j,j}. \tag{S2}$$

To apply these results to distribution n' of the $(M, N+1)$ ensemble we replace N with $N+1$:

$$\bar{k}_{\mathbf{n}'} = \frac{1}{N(N+1)} \sum_{i=1}^{\infty} \sum_{j=1}^{\infty} n'_i (n'_j - \delta_{ij})\, k_{ij} \tag{S3}$$

$$= \frac{2}{N(N+1)} \sum_{i=1}^{\infty} \sum_{j=1}^{i} \frac{n'_{i-j}(n'_j - \delta_{i-j,j})}{1+\delta_{i-j,j}}\, k_{i-j,j}. \tag{S4}$$

Equation (S3) is Eq. (10) of the text.

We may now confirm that the transition probability $P_{\mathbf{n}'\to\mathbf{n}}$ is properly normalized:

$$\sum_{\text{all } i,j \text{ in } \mathbf{n}'} P_{\mathbf{n}'\to\mathbf{n}} = \frac{2}{N(N+1)\bar{k}_{\mathbf{n}'}} \sum_{i=1}^{\infty}\sum_{j=1}^{\infty} \frac{n'_{i-j}(n'_j - \delta_{i-j,j})}{1+\delta_{i-j,j}}\, k_{i-j,j} = 1.$$

The final result on the RHS follows from Eq. (S4).

B. Derivation of Recursions, Eqs. (12) and (14)

First write Eq. (11) in the form

$$\frac{\Omega_{M,N+1}}{\Omega_{M,N}} = \frac{M-N}{N}\frac{A}{\langle k_{M,N+1}\rangle}, \tag{S5}$$

$$A = \frac{1}{M-N}\sum_{i=2}^{\infty} n_i \sum_{j=1}^{i-1} \frac{k_{i-j,j}}{\bar{k}_{\mathbf{n}'}/\langle k_{M,N+1}\rangle}\frac{W(\mathbf{n}')}{W(\mathbf{n})}. \tag{S6}$$

Since the LHS in Eq. (S5) is independent of distribution $\mathbf{n}$, A must be an (intensive) ensemble property. In the thermodynamic limit the bias of parents and offsprings satisfies the homogeneity condition [1]

$$\log W(\mathbf{n}) = \sum_i n_i \log \tilde{w}_i \tag{S7}$$

from which we obtain

$$\frac{W(\mathbf{n}')}{W(\mathbf{n})} = \frac{\tilde{w}_{i-j}\tilde{w}_j}{\tilde{w}_i}. \tag{S8}$$

We use the above result to express Eq. (S6) in the form

$$A = \frac{1}{M-N}\sum_{i=2}^{\infty} n_i (i-1)\, a_i \tag{S9}$$

$$a_i = \frac{1}{i-1}\sum_{j=1}^{i-1} \frac{k_{i-j,j}}{\bar{k}_{\mathbf{n}'}/\langle k_{M,N+1}\rangle}\frac{\tilde{w}_{i-j}\tilde{w}_j}{\tilde{w}_i}. \tag{S10}$$

Here $\mathbf{n}'$ is the parent distribution that produces the offspring distribution $\mathbf{n}$ by aggregation of the cluster pair $(i-j)+(j)$. In the thermodynamic limit ($\mathbf{n}\to\tilde{\mathbf{n}}$), the ratio $\bar{k}_{\mathbf{n}'}/\langle k_{M,N+1}\rangle$ is independent of the individual parent and becomes a function of $i-j$ and j. The j summation then produces a result, let's call it a_i, that is a function of i, M and N. The set $(a_1, a_2\cdots)$ must satisfy Eq. (S9) for all distributions $\mathbf{n}$ in the vicinity of the MPD. This can only be if $a_i =$ const. It follows $A = a_i =$ const. Now, from Eq. (S6) we note that the numerical value of A multiplies all W by that factor. Treating Eq. (S10) as a recursion for

$\tilde{w}_i$, we see that the numerical value of a multiplies $\tilde{w}_i$ by the factor a^{i-1}. Since $A = a$, and Eq. (S7) must be satisfied, we conclude $A = a = 1$. Equation (S5) now gives

$$\frac{\Omega_{M,N+1}}{\Omega_{M,N}} = \frac{M-N}{N}\frac{1}{\langle k_{M,N+1}\rangle}, \tag{S11}$$

which is Eq. (12) of the text. Solving Eq. (S6) for $W(\mathbf{n})$ we also have

$$W(\mathbf{n}) = \frac{\langle k_{M,N+1}\rangle}{M-N}\sum_{i=2}^{\infty} n_i \sum_{j=1}^{i-1}\frac{k_{i-j,j}}{\bar{k}_{\mathbf{n}'}}\, W(\mathbf{n}'). \tag{S12}$$

which is Eq. (14) of the text.

C. Solution for the Product Kernel

With $k_{ij} = ij$, the mean kernel in a distribution $\mathbf{n}$ of the (M, N) ensemble is

$$\bar{k}_{\mathbf{n}} = \frac{\sum_i \sum_j ijn_in_j}{N(N-1)} - \frac{\sum_i i^2 n_i}{N(N-1)} \to \frac{\sum_i \sum_j ijn_in_j}{N(N-1)}.$$

Asymptotically,

$$\bar{k}_{\mathbf{n}} \to \langle k_{M,N}\rangle \to \left(\frac{M}{N}\right)^2.$$

Applying this result to Eq. (11) we obtain

$$\Omega^{\text{prod}}_{M,N} = \left(N!\,\frac{M^{M-N}}{M!}\right)^2 \binom{M-1}{N-1}.$$

The parameters β and q are obtained from the discrete version of the derivatives in Eqs. (4), (5):

$$\begin{aligned}\beta = \log\frac{\Omega_{M+1,M}}{\Omega_{M,N}} &= \log\left(\frac{M^{-2(M-N)+1}(M+1)^{2(M-N)}}{M-N+1}\right)\\ &\to 1-\frac{N}{M} - 2\log\left(1-\frac{N}{M}\right),\end{aligned} \tag{S13}$$

and

$$q = \frac{\Omega_{M,N+1}}{\Omega_{M,N}} = \frac{(N+1)^2(M-N)}{M^2N} \to \frac{N}{M}\left(1-\frac{N}{M}\right). \tag{S14}$$

Equations (S13), (S14) are Eqs. (16), (17), of the text.

From Eq. (S10) with $a_i = 1$ and $\bar{k}_{\mathbf{n}'}/\langle k_{M,N+1}\rangle = 1$ we obtain a recursion for w_i:

$$\tilde{w}_i = \frac{1}{i-1}\sum_{j=1}^{i-1}(i-j)j\tilde{w}_{i-j}\tilde{w}_j, \tag{S15}$$

whose inversion gives [2]

$$\tilde{w}_i = 2\frac{(2i)^{i-2}}{i!}. \tag{S16}$$

The last result is Eq. (18) of the text.

[1] Matsoukas, T. Statistical thermodynamics of clustered populations. *Phys. Rev. E* **90**, 022113 (2014).

[2] Leyvraz, F. Scaling theory and exactly solved models in the kinetics of irreversible aggregation. *Physics Reports* **383**, 95–212 (2003).
